# Baicalein protects renal tubular epithelial cells againsthypoxia-reoxygenation injury

**DOI:** 10.1080/0886022X.2018.1532910

**Published:** 2018-11-01

**Authors:** Chun Chen, Chudan Cai, Hanfei Lin, Weidai Zhang, Yanqiang Peng, Kefei Wu

**Affiliations:** a Department of Traditional Chinese Medicine, The First Affiliated Hospital of Shantou University Medical College, Shantou, China;; b Department of Nephrology, The First Affiliated Hospital of Shantou University Medical College, Shantou, China

**Keywords:** Baicalein, oxidative stress, inflammation, hypoxia-reoxygenation, renal tubular epithelial cell

## Abstract

**Background:** To investigate the protective effects and mechanism of baicalein (BAI), a naturally occurring flavonoid, against hypoxia-reoxygenation (HR) injury in renal tubular epithelial cells (HK-2).

**Methods:** Cultured human renal proximal tubular cell line HK-2 was exposed to 24 h of hypoxia (5% CO_2_, 1% O_2_, and 94% N_2_), followed by 12 h of reoxygenation (5% CO_2_, 21% O_2_, and 74% N_2_). HK-2 cells were divided into three groups: control, HR, and HR-BAI (0.3 µg/ml). Reactive oxygen species (ROS) were measured and cell apoptosis was analyzed by flow cytometry and morphology. ELISAs were performed to determine the levels of IL-1, intercellular adhesion molecule-1 (ICAM-1), and monocyte chemotactic protein-1 (MCP-1). IL-1β, ICAM-1, and MCP-1 mRNA levels were determined by real-time quantitative PCR.

**Results:** HK-2 cells that underwent HR exhibited increases in IL-1β expression by 0.94%, ROS by 0.59%, ICAM-1 expression by 0.8%, and MCP-1 expression by 1.2%. Moreover, HK-2 cell apoptosis was increased after HR (*p* < .05). Compared with the HR group, BAI treatment reduced the elevation of oxidative stress (ROS) by 0.76%, as well as HR-mediated induction of IL-1β and apoptosis of HK2 cells. Protein and mRNA levels of ICAM-1 and MCP-1 were also reduced.

**Conclusions:** BAI protects renal tubular epithelial cells from HR injury by reducing inflammatory cytokine expression and oxidative stress.

## Introduction

Acute kidney injury (AKI) is a frequently severe clinical emergency. AKI has a high risk of death and progression to chronic kidney disease, especially in critically ill patients, which has become a worldwide public health problem [1,2]. The pathogenesis and prognosis of AKI have been studied more extensively in the past decade, which has revealed a series of biomarkers, the optimal dose and intensity of renal replacement therapy, and the effects of fluid management. Effective use of various drugs, including dopamine, atrial natriuretic peptide, and insulin-like growth factor, has been determined in animal trials, but not in clinical studies [3–6]. About 20–30% of AKI patients develop chronic kidney injury and eventually succumb to end stage kidney disease. Therefore, more efficient and practical methods are needed for timely prevention and a cure.

Many heterogeneous pathological factors may lead to AIK. The predominant mechanism responsible for AKI is acute renal ischemia-reperfusion injury [7–9]. Recent studies show that oxygen free radicals, inflammatory mediators, such as interleukin-1 (IL-1), intercellular adhesion molecule-1 (ICAM-1), and monocyte chemotactic protein-1 (MCP-1), inflammatory cell infiltration, and especially oxygen free radicals are related to renal ischemia-reperfusion injury [10–13]. A study has found that antioxidants reduce oxidative stress, which have become the focus of prevention and treatment of various oxidative stress-related diseases [14].

Baicalein (BAI, 5,6,7-trihydroxy-2-phenyl-4H-1-benzopyran-4-one) is a natural flavonoid with anti-oxidant and anti-inflammatory effects [5–7]. It is an active component in *Scutellaria baicalensis Georgi*, which is cultivated in Siberia, Mongolia, the Russian Far East, China, and Korea, and one of the 50 fundamental herbs used in traditional Chinese medicine [15]. BAI (huang qin in Chinese) inhibits cardiomyocyte apoptosis, inflammatory responses, and oxidative stress in the heart after ischemia-reperfusion injury [13]. In our previous study, we found that administration of BAI after AKI alleviates renal ischemia-reperfusion injury and promotes recovery of renal function in rats. However, the effects of BAI on AKI and the molecular mechanisms remain unclear. We used a human renal proximal tubular cells (HK-2) to establish a model of hypoxia-reoxygenation (HR) injury to simulate acute renal ischemia-reperfusion injury. We focused on oxidative stress, inflammation, and apoptosis *in vitro* to explore the effects and mechanisms of BAI in HR injury of HK-2 cells.
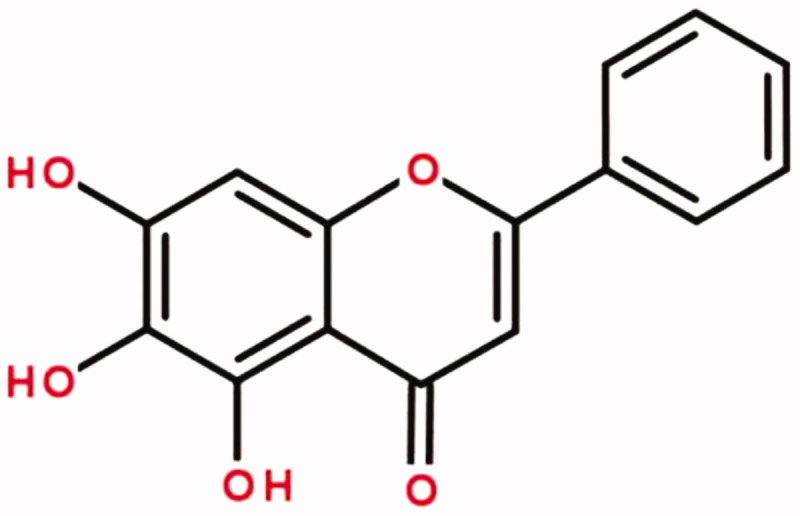
The structural formula of BAI

## Materials and methods

### Reagents

The human renal proximal tubular cell line HK-2 was obtained from the American Type Culture Collection (Manassas, VA, USA). Dulbecco’s modified Eagle’s medium (DMEM)/F12, fetal bovine serum (FBS), trypsin, Hank’s buffered saline, and Roswell Park Memorial Institute 1640 (RPMI-1640) medium were purchased from Gibco Technologies (Logan, UT, USA). BAI was purchased from Santa Cruz Biotechnology (Santa Cruz, CA). An Annexin V-fluorescein isothiocyanate (FITC) apoptosis detection kit was obtained from Biovision (Milpitas, CA, USA). ICAM-1, MCP-1, and IL-1 enzyme-linked immunosorbent assay (ELISA) kits were purchased from Baoman Biotechnology Co., LTD (Shanghai, China). Antibodies against ICAM-1, MCP-1, and β-actin were purchased from Abcam (Cambridge, UK).

### Cell culture

Passage 2 or 4 HK2 cells were cultured in DMEM/F12 supplemented with 10% heat-inactivated FBS at 1 × 10^6^ cells per well in 6-well culture plates at 37 °C with 5% CO_2_ for 24 h. Various doses of BAI were added at 2 h before exposure to HR. Cells were randomly divided into three groups: (1) Control: cells were incubated in normoxic conditions (5% CO_2_, 21% O_2_, and 74% N_2_) without BAI treatment; (2) HR: cells were exposed to 24 h of hypoxia (5% CO_2_, 1% O_2_, and 94% N_2_), followed by 12 h of reoxygenation (5% CO_2_, 21% O_2_, and 74% N_2_); (3) HR-BAI: cells pretreated with BAI (0.3 µg/ml) were exposed to 24 h of hypoxia, followed by 12 h of reoxygenation.

### Cytotoxicity assay of HK-2 cells

A 3–(4,5)-dimethylthiahiazol (-z-y1)-3,5-di-phenytetrazoliumromide (MTT) assay was used to analyze the cytotoxicity of BAI in HK-2 cells. Cells were cultured in 96-well plates (1 × 10^4^ per well) with DMEM alone or treated with BAI (0.1, 0.2, 0.3, 0.4, and 0.5 µg/ml) [16,17] for 24 h. After removing the medium, MTT was dissolved in PBS (5 mg/mL) and added to each well, followed by incubation for 4 h. The cells were then dissolved in DMSO. Absorbance was measured by an ELISA analyzer (Thermo Fisher Scientific, Waltham, MA) at 490 nm. Wells without cells were considered as the blank. Results were expressed as percentages of control. The cell viability of each group was calculated by the following formula [18], and the optimal protective concentration of BAI in cells was selected.

Cell proliferation rate (%)=(OD for model and BAI control samples–OD for blank)/(OD for control–OD for blank)×100

### Measurement of ROS by flow cytometry (FCM)

ROS in HK-2 cells was detected by FCM using the oxidation sensitive fluorescent probe dichlorofluorescein (DCFH) [19]. Thirty minutes before cell collection, 0.1 µM DCFH was added to the cells, and then the cells were centrifuged at 1000 rpm for 10 min and washed in cold PBS three times. Then, the cells were resuspended in 0.5 mL stationary buffer (0.3 mL PBS and 0.2 mL of 4% paraformaldehyde) at 4 °C while protected from light. The fluorescence intensity of the cells was detected by FCM at 488 nm. The experiments were repeated three times.

### Morphological detection and measurement of cell apoptosis by FCM

HK-2 cell apoptosis were determined by Hoechst 33258 staining and morphology under an inverted microscope (Eclipse TS100, Nikon, Japan). The HK-2 cells were cultured in 6-well plates and treated as described above. Then, the cells were washed with PBS for three times, fixed with 4% paraformaldehyde for 10 min, washed with PBS, and stained with Hoechst 33258 for 10 min. The nuclear morphology of cells was visualized by fluorescence microscopy (Eclipse TE300, Nikon, Japan) at 460 nm. Cells that had nuclear fragmentation and chromatin condensation with blue fluorescence were considered as apoptotic cells [20]. The experiments were repeated three times. Annexin V-FITC/PI staining was used to examine cell apoptosis by laser scanning confocal microscopy (Olympus, Japan). The results were analyzed using CellQuest software [21]. The experiments were repeated three times.

### Detection of IL-1, ICAM-1, and MCP-1 levels in HK-2 cells by ELISAs

IL-1, ICAM-1, and MCP-1 levels in conditioned medium of HK-2 cells were detected using corresponding ELISA kits (Baoman Biotechnology). The supernatant liquid in each well were collected in corresponding centrifuge tube and then centrifuged for 15 min at 3000 rpm/min. The clear supernatant liquid was transferred to a new corresponding tube as samples. Standard liquid was added in a 96-well plate and then diluted to the concentration of full, 0.5 times, 0.25 times,0.125 times and 0.0625 times and made sure there was 50 μL liquid per well. We also set a blank control without any treatment and then each standard formulas were calculated after detected in corresponding wavelengths. 50 μL samples were added in this 96-well plate with repeated wells for three times. Absorbance at 450 nm was determined using a Bio-Rad microplate reader. The experiments were repeated three times. Finally, the OD data were calculated with the standard formula and analyzed statistically.

### Real-time quantitative PCR (RT-PCR) of IL-1, ICAM-1, and MCP-1 mRNAs in HK-2 cells

Total RNA of HK-2 cells was extracted with RNA extraction reagent (TaKaRa Bio, Shiga, Japan). Then, cDNA was synthesized using a TaKaRa Bio Reverse Transcription kit, according to the manufacturer instructions. First-strand cDNA was used for quantitative PCR using a 7500 Real-Time PCR System (Applied Biosystems) with GAPDH as the internal reference. Amplification and melting curves were obtained using the 2^-ΔΔCt^ method. The experiments were repeated three times.

**Table:** 

mRNA	Sense primer	Anti-sense primer
IL-1beta	5′-ATAGCAGCT TTCGACAGTGAG-3′	5 ′-GTCAACTATGTCCCGACCATT-3′
MCP-1	5′-ACGCTTCTGGGCCTGTTGTTCA-3′	5′-TGGGGCATTAACTGCATCTGGCT-3′
ICAM-1	5′-GCGACCACGGAGCCAATTTCTCAT-3′	5′-TCAGGACCCTAGTCGGAAGATCGAA-3′
GAPDH	5′-GGAGCGAGACCCCACTAACA-3′	5′-GGCGGAGATGATGACCCTT-3′

### Statistical analysis

Figures were prepared by GraphPad Prism 5. Statistical analysis was performed by SPSS 22.0 software. Data are expressed as means ± standard deviation (SD). Differences among groups were analyzed using one-way ANOVA. The least-square difference test or Dunnett’s T3 test (equal variance not assumed) was used to ascertain significant differences among groups. Values of *p* < .05 were considered to be significant.

## Results

### BAI improves the viability of HR-treated HK-2 cells

**Figure 1.**
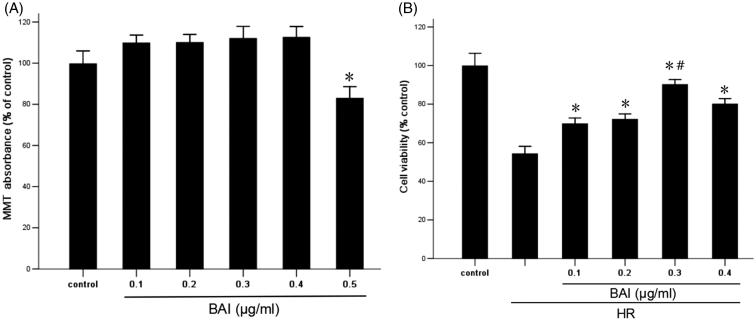
(A) Effect of BAI at various concentration on the proliferation of HK-2 cells under normoxic conditions. Results are expressed as the mean ± SD (*n* = 9). *p < .05 vs. control. (B) Effect of BAI at various concentration on the viability of HR-treated HK-2 cells. Results are expressed as the mean ± SD (*n* = 9). *p < .05 vs. HR group without treatment; #*p* < .05 vs. other HR + BAI groups.

As shown in Figure 1(A), under the normoxic condition, the effect of BAI (0.5 µg/ml) on cell proliferation was decreased significantly compared with the control group, indicating that the cytotoxicity of HK-2 cells was enhanced significantly (*p* < .05). Therefore, after HR treatment, four doses of BAI were applied to HK-2 cells. Cell viability was measured by MTT assays. As shown in Figure 1(B), after HR treatment, cell viability was decreased significantly (*p* < .05). However, in the BAI intervention group, cell viability was increased and 0.3 µg/ml BAI had the highest effect. Therefore, we used 0.3 µg/ml BAI for subsequent experiments.

### BAI reduces HR-induced IL-1β levels in HK-2 cells

**Figure 2.**
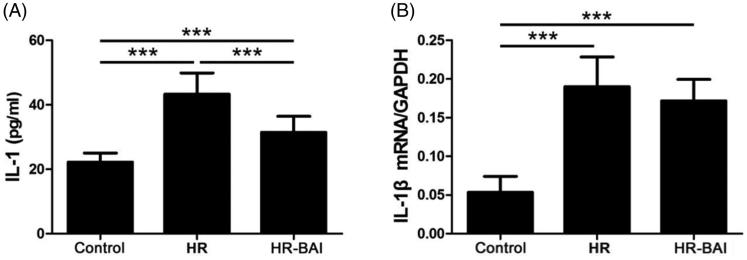
BAI reduces HR-induced IL-1β levels in HK-2 cells. (A) Determination of IL-1β levels in culture supernatants by ELISA. (B) IL-1β mRNA levels in HK-2 cells. ***p < .001.

IL-1 is a proinflammatory cytokine secreted mainly by macrophages as well as many kinds of nucleated cells, which aggravates inflammation following with HR injury. The IL-1β level in the culture supernatant was higher in the HR group (43.33 ± 6.55 pg/ml) than in the control group (22.23 ± 1.14 pg/ml, *p* < .05). However, the IL-1 level in the HR-BAI group (31.47 ± 4.93 pg/ml) was obviously lower compared with that in the HR group (*p* < .01; Figure 2(A)). IL-1β mRNA expression was also increased by HR exposure (0.19 ± 0.04) compared with the control group (0.05 ± 0.02). However, BAI treatment decreased the IL-1 mRNA level (0.17 ± 0.03) compared with the HR group, although the difference was not significant (*p* > .05; Figure 2(B)). Therefore, BAI inhibited the release of IL-1 from HK-2 cells after HR injury.

### BAI reduces ICAM-1 expression in HR-exposed HK-2 cells

**Figure 3.**
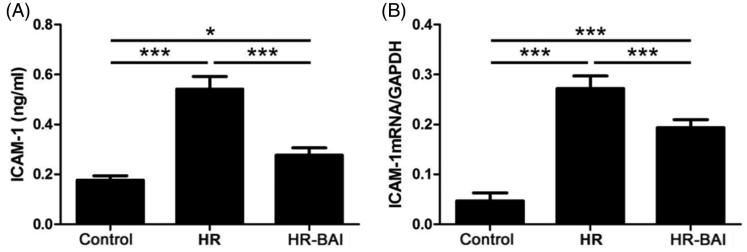
BAI reduces ICAM-1 levels in HK-2 cells following HR. (A) Determination of ICAM-1 levels in culture supernatants by ELISA. (B) ICAM-1 mRNA levels in HK-2 cells. ***p < .001, *p < .05.

The ICAM-1 level in the culture supernatant was increased in the HR group (0.54 ± 0.05 ng/ml) and HR-BAI group (0.28 ± 0.03 ng/ml) compared with the control group (0.18 ± 0.02 ng/ml). The differences between all groups were statistically significant (*p* < .05). BAI reduced the ICAM-1 level in HR-exposed HK-2 cells (*p* < .01; Figure 3(A)). ICAM-1 mRNA was increased by HR exposure (0.27 ± 0.03) compared with the control group (0.05 ± 0.02). BAI treatment decreased ICAM-1 mRNA levels (0.19 ± 0.02) compared with the HR group, and the difference was statistically significant (*p* < .01; Figure 3(B)).

### BAI decreases MCP-1 levels in HR-exposed HK-2 cells

**Figure 4.**
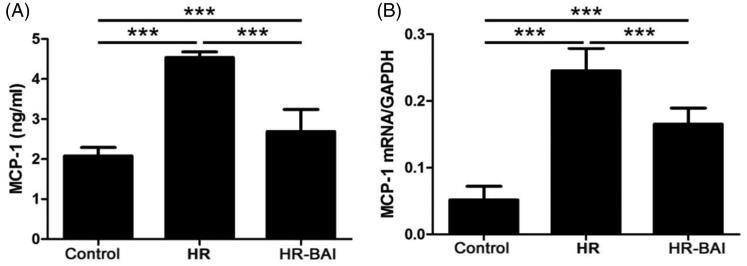
BAI reduces MCP-1 levels in HR-exposed HK-2 cells. (A) Determination of MCP-1 levels in culture supernatants by ELISA. (B) MCP-1 mRNA level in HK-2 cells. ***p < .001. BAI reduces the ROS level in HK-2 cells after HR.

Compared with the control group (2.08 ± 0.09), MCP-1 levels in the culture supernatant was increased in the HR group (4.54 ± 0.14) and HR-BAI group (2.69 ± 0.55, *p* < .01). However, BAI decreased the MCP-1 level in HR-exposed HK-2 cell culture supernatants (*p* < .01; Figure 4(A)). Furthermore, HR treatment upregulated the MCP-1 mRNA level (0.25 ± 0.03) compared with the control group (0.05 ± 0.02, *p* < .01) and inhibited the HR-mediated increase (0.17 ± 0.02, *p* < .01; Figure 4(B)).

**Figure 5.**
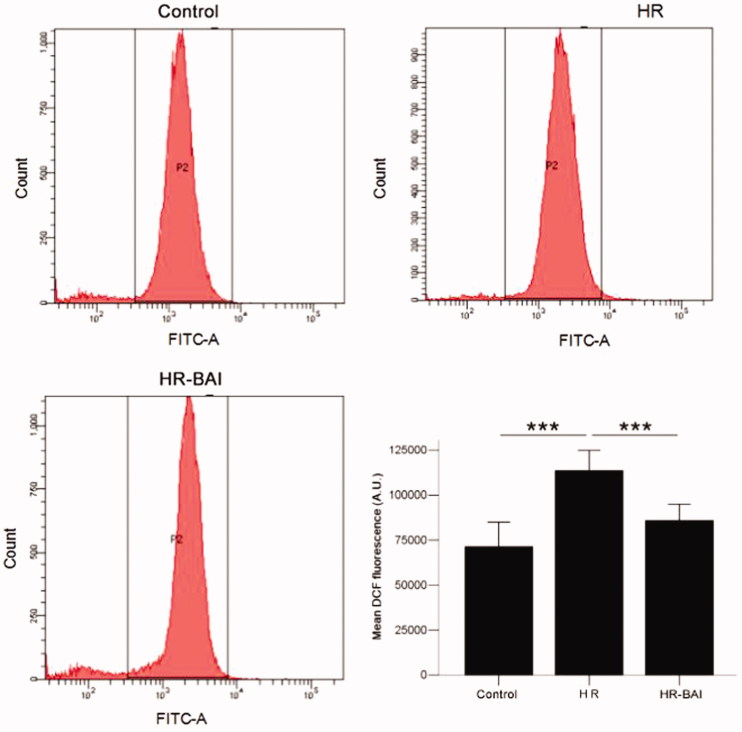
BAI reduces elevated ROS levels in HK-2 cells following HR. ***p < .001.

The intracellular ROS level in HK-2 cells was evaluated by measuring the fluorescence intensity of DCFH by FCM. After HR treatment (113653.00 ± 5669.18 A.U.), the ROS level was increased in HK-2 cells (control group: 71378.56 ± 6833.56 A.U., *p* < .01). However, the HR-induced increase in ROS level was dramatically reduced by BAI treatment (85943.22 ± 4514.47 A.U., *p* < .01; Figure 5). These results indicate that BAI decreases the ROS level after HR treatment to protect HK-2 cells from oxidative damage.

### BAI decreases apoptosis after HR

**Figure 6.**
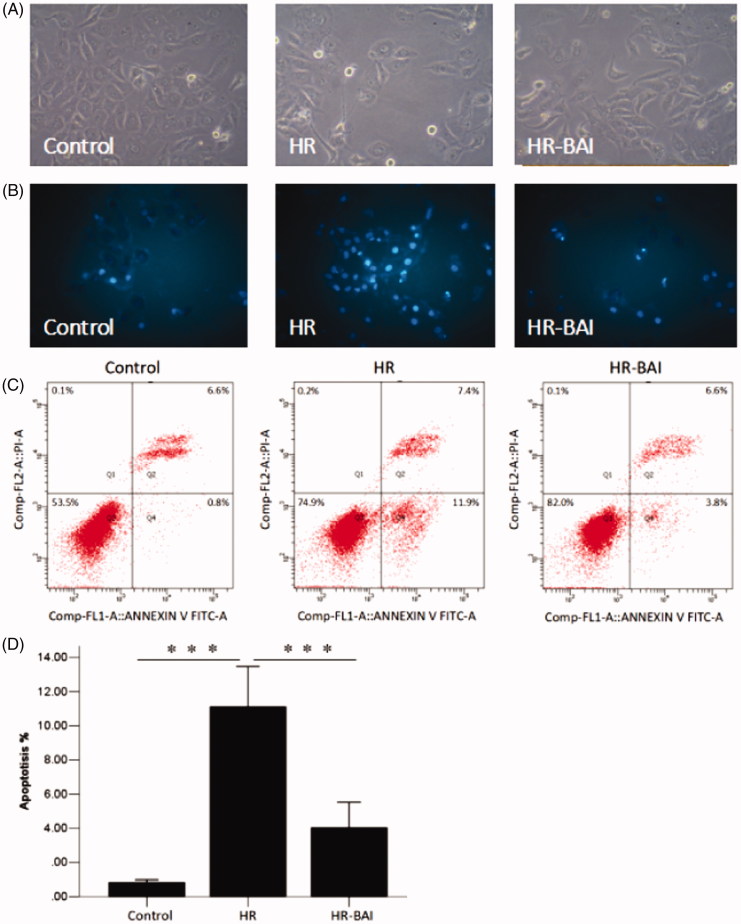
BAI treatment decreases cell apoptosis after HR. (A) Observation under an inverted microscope (×100). HK-2 cells in the HR group showed cytoplasmic blebbing, necrosis, and nuclear pyknosis, whereas BAI protected cells from these events. (B) Observation under a fluorescence inverted microscope (×400). The nuclei of apoptotic cells stained with Hoechst 33258 showed blue fluorescence. Control and BAI groups showed less blue fluorescence than the HR group. This was the typical morphological changes of apoptosis. (C) Flow cytometry of HK-2 cells following the various treatments. (D) Histogram of HK-2 cell apoptosis. ***p < .001.

Compared with the control group (0.82 ± 0.07%), cell apoptosis was increased in the HR group (11.11 ± 1.18%) and HR-BAI group (4.03 ± 0.74%, *p* < .05). However, BAI treatment reduced HK-2 cell apoptosis compared with the HR group (*p* < .05; Figure 6).

## Discussion

In our previous study using a renal ischemia reperfusion injury rat model, we found that BAI decreased accumulation of advanced oxidation protein products and malondialdehyde levels, inhibited the expression of inflammatory factors, and markedly improved renal functions [22]. To better understand how BAI alleviates renal ischemia reperfusion injury, we examined the cytoprotective effects of BAI on HK-2 cells following exposure to HR. It was reported that oxygen free radicals, cellular adhesion molecules and inflammatory factors may intensify renal injury after ischemia reperfusion [2–4]. In recent years, BAI has been reported to possess antioxidant and free radical scavenging activities [5,6,10]. To our knowledge, this is the first study to demonstrate that BAI protects against HR injury in human renal tubular epithelial cells through oxidative stress and inflammatory pathways.

Hypoxia promotes renal ischemic injury via increasing oxidative stress . Furthermore, increasing ROS after reoxygenation and decreasing antioxidants lead to renal injury [23–25].

This is consistent with acute kidney injury patients showing higher oxidative stress [26]. ROS, synthesized after reperfusion in renal proximal tubules were considered to be major contributing factors to ischemia/reperfusion injury [27,28]. Oxygen free radicals inhibit renal reperfusion directly, and even enhance inflammatory responses as well as apoptosis [29]. Furthermore, the toxic reactive intermediates induce cellular oxidants and damage cellular components such as proteins, the genome, and membranes, leading to renal damage [14]. In conclusion, a therapy to inhibit generation of ROS and free radicals has become important for the prevention of AKI.

In HK-2 cells following HR injury, we observed elevation of ROS, reflecting an increased level of oxidative stress. HR likely induces generation of oxygen free radicals that cause cell damage. We observed that cell apoptosis increased under HR injury and coincided with the elevation of ROS.

Apoptosis induced by oxygen free radicals enhances HR-induced acute renal failure. In HK-2 cells subjected to HR, ICAM-1, MCP-1, and IL-1 protein expression levels were increased, while ICAM-1 and MCP-1 mRNA expression levels were increased correspondingly. High expression of ICAM-1, MCP-1, and IL-1 indicates an inflammatory response during HR. Additionally, we observed that BAI induced decreases in ICAM-1, MCP-1, and IL-1 expression, which was consistent with the decreased apoptosis of BAI-treated HK-2 cells, revealing that inflammatory responses following hypoxia reperfusion aggravate acute renal failure. These results are consistent with our previous *in vitro* study [22].

In our study, we measured cellular ROS levels, as well as cell survival and apoptosis. BAI reduced HR-induced apoptosis, increased the survival of HR-exposed cells, and suppressed ROS generation. These results demonstrate that BAI plays a renal protective role through decreasing the production of oxygen free radicals in cells and inhibiting HR-induced apoptosis.

We next investigated whether the inhibitory effects of BAI on HR-induced apoptosis were mediated through decreasing the inflammatory response. HR-exposed HK-2 cells administered BAI displayed reduced levels of ICAM-1, MCP-1, and IL-1 proteins, and lower ICAM-1 and MCP-1 mRNA expression levels than the HR group. Apoptosis was decreased in the BAI-HR group in comparison with the HR group. These results demonstrate that HR induces an oxidative stress response that stimulates the production of ROS and triggers inflammatory response-meditated apoptosis. In a previous study, we showed that administration of BAI to rats after AKI alleviates renal ischemia reperfusion injury and promotes recovery of renal functions. BAI decreases intracellular oxidative stress and inhibits the production of oxygen free radicals to alleviate lipid peroxidation, cytokines release, apoptosis, and inflammatory responses of injured cells [22]. The study of Lai CC showed that BAI significantly attenuates kidney injury induced by myocardial ischemia and reperfusion. The possible mechanisms might be related to the inhibition of apoptosis, through the reduction of tumor necrosis factor-α, IL-1, IL-6 in the kidneys [30]. The discovery of Sahu BD suggested that BAI ameliorates cisplatin-induced renal injury through up-regulation of antioxidant defense mechanisms and down regulation of the MAPKs and NF-κB signaling pathways [31]. Our *in vitro* experiments provide further direct evidence that administration of BAI before reoxygenation of cells protects against HR via antioxidant, anti-apoptotic, and anti-inflammatory effects.

## Conclusions

BAI protects against HR injury in renal tubular epithelial cells via anti-inflammatory effects and reducing oxidation stress.
